# Shifts in leaf litter breakdown along a forest–pasture–urban gradient in Andean streams

**DOI:** 10.1002/ece3.2257

**Published:** 2016-06-17

**Authors:** Carlos Iñiguez‐Armijos, Sirkka Rausche, Augusta Cueva, Aminael Sánchez‐Rodríguez, Carlos Espinosa, Lutz Breuer

**Affiliations:** ^1^ Departamento de Ciencias Naturales Universidad Técnica Particular de Loja (UTPL) San Cayetano Alto s/n 1101608 Loja Ecuador; ^2^ Institute for Landscape Ecology and Resources Management (ILR) Research Centre for Biosystems Land Use and Nutrition (IFZ) Justus Liebig University Giessen Heinrich‐Buff Ring 26 35392 Giessen Germany; ^3^ Centre for International Development and Environmental Research Justus Liebig University Giessen Giessen Germany

**Keywords:** Andean streams, aquatic decomposer communities, land‐use change, leaf litter breakdown, nutrient enrichment, pH, water temperature

## Abstract

Tropical montane ecosystems of the Andes are critically threatened by a rapid land‐use change which can potentially affect stream variables, aquatic communities, and ecosystem processes such as leaf litter breakdown. However, these effects have not been sufficiently investigated in the Andean region and at high altitude locations in general. Here, we studied the influence of land use (forest–pasture–urban) on stream physico‐chemical variables (e.g., water temperature, nutrient concentration, and pH), aquatic communities (macroinvertebrates and aquatic fungi) and leaf litter breakdown rates in Andean streams (southern Ecuador), and how variation in those stream physico‐chemical variables affect macroinvertebrates and fungi related to leaf litter breakdown. We found that pH, water temperature, and nutrient concentration increased along the land‐use gradient. Macroinvertebrate communities were significantly different between land uses. Shredder richness and abundance were lower in pasture than forest sites and totally absent in urban sites, and fungal richness and biomass were higher in forest sites than in pasture and urban sites. Leaf litter breakdown rates became slower as riparian land use changed from natural to anthropogenically disturbed conditions and were largely determined by pH, water temperature, phosphate concentration, fungal activity, and single species of leaf‐shredding invertebrates. Our findings provide evidence that leaf litter breakdown in Andean streams is sensitive to riparian land‐use change, with urban streams being the most affected. In addition, this study highlights the role of fungal biomass and shredder species (*Phylloicus*; Trichoptera and *Anchytarsus*; Coleoptera) on leaf litter breakdown in Andean streams and the contribution of aquatic fungi in supporting this ecosystem process when shredders are absent or present low abundance in streams affected by urbanization. Finally, we summarize important implications in terms of managing of native vegetation and riparian buffers to promote ecological integrity and functioning of tropical Andean stream ecosystems.

## Introduction

Changes in the structure and functioning of stream ecosystems are closely related to riparian vegetation and land uses (Allan 2004). The principal mechanisms by which land use influences stream variables controlling aquatic communities closely related to ecosystem processes such as leaf litter breakdown have been well studied in temperate streams (e.g., Danger and Robson 2004; Paul et al. 2006; McKie and Malmqvist 2009; Young and Collier 2009; Hladyz et al. 2011) and in some lowland tropical streams (e.g., Silva‐Junior and Moulton 2011). For instance, the conversion of forest to pasture or urbanization can alter stream flow and pH, increase water temperature and nutrient concentrations, and reduce invertebrate densities (Paul and Meyer 2001; Allan 2004). In turn, changes in these stream variables can affect the dynamics of leaf litter breakdown in different manners. Increased stream flow (current velocity; discharge; storm runoff) caused by altered channel dynamics, and impervious surface can produce physical abrasion of leaves in urban streams (Paul et al. 2006). Low pH can reduce the diversity of decomposer communities affecting directly the organic matter processing by decreasing breakdown rates (Dangles and Chauvet 2003; Dangles et al. 2004; Petrin et al. 2007). Increased water temperature caused by light penetration because of riparian clearing, and increased nutrient concentrations (i.e., N, P) caused by cattle grazing in pasture land streams and by sewage in urban streams, can synergistically stimulate fungal activity accelerating breakdown rates (Chadwick et al. 2006; Imberger et al. 2008; Hladyz et al. 2010; Ferreira and Chauvet 2011). On the other hand, increased water temperature and nutrients can reduce the availability of dissolved oxygen (Allan 2004), which in turn can diminish the activity of leaf associated fungi and reduce or eliminate leaf‐shredding invertebrates (Couceiro et al. 2006; Medeiros et al. 2009). Furthermore, the replacement of native riparian vegetation by pasture land or urbanization can cause a decrease in organic matter input and a reduction in litter quality (Paul and Meyer 2001; Allan 2004). Therefore, the shredder density and fungal activity can be diminished by food limitation and poor litter quality, affecting leaf litter breakdown (Lecerf et al. 2005; Wantzen and Wagner 2006; Graça et al. 2015). These different responses of stream variables, aquatic communities, and leaf litter breakdown to changes in land use may be attributed to different types and intensities of human land use being compared across studies and to altitudinal and latitudinal factors present at different ecoregions (Young et al. 2008). Therefore, it is important that we study the effects of land use in Andean streams and in other tropical montane rainforest streams because alterations in leaf litter breakdown have barely been studied in these montane ecosystems.

The Andes are recognized for providing essential ecosystem services including carbon storage, hydropower, and water supply (Buytaert et al. 2011; Breuer et al. 2013; Knoke et al. 2014). They are also considered as a biodiversity hot spot (Mittermeier et al. 1998; Myers et al. 2000). Moreover, although the Andes represent a minor portion of the Amazon basin, the contribution of their streams in terms of water discharge and nutrients to lowlands is significantly large (Lujan et al. 2013). The Andean ecosystems are critically threatened due to rapid land‐use change during the last few decades (Sierra et al. 2002; Peters et al. 2013; Balthazar et al. 2015). The relationships between land‐use and stream physico‐chemical variables, biological communities, and ecosystem processes in Andean streams have been poorly studied despite the rapid land‐use change documented over large areas along the Andes (Etter et al. 2006; Armenteras et al. 2011; Tapia‐Armijos et al. 2015). In addition, anthropogenic activities appear to be expanding more rapidly in Andean watersheds than in other areas of the Amazon basin (Lujan et al. 2013). Historically, the conversion of native forest to pasture for livestock has been the largest and most destructive change in land use for Andean ecosystems (Armenteras et al. 2003; Thies et al. 2014). This has been followed by urban development which is mostly concentrated in inter‐Andean valleys (Sarmiento 2002). In consequence, it is likely that the hydrologic alterations, disruption in riparian‐stream linkage, nutrient enrichment, and pollution observed in Andean streams in recent decades are an effect of land‐use change and associated anthropogenic activities (Lujan et al. 2013). Few studies carried out in tropical Andean streams have demonstrated that deforestation affects the ecological conditions by decreasing water quality and altering benthic macroinvertebrate assemblages (Bücker et al. 2010; Iñiguez‐Armijos et al. 2014). Similarly to other montane freshwater (Taylor and Chauvet 2014; Astudillo et al. 2016), the conversion of forest to pasture can alter decomposer communities and reduce the processing rates of organic matter (Encalada et al. 2010). Therefore, more research is needed to understand the influence of land use on key ecosystem processes such as leaf litter breakdown in these tropical montane streams.

Leaf litter breakdown is a key stream process in particular for streams where organic matter inputs represent an important source of energy and nutrients (Graça 2001). In those streams, leaf litter breakdown is driven by microbial activity, mainly microfungi, and by leaf‐shredding invertebrates, but also is the result of physical abrasion and leaching (Graça 2001; Hieber and Gessner 2002). It is well known that aquatic communities play a significant role during this ecosystem process. Once in the stream, organic matter is quickly colonized by fungi that degrade leaf constituents and thus enhance leaf litter breakdown (Gessner et al. 2010). This fungal colonization increases the palatability of leaves and reduces the leaf toughness that facilitates the feeding by shredder invertebrates promoting leaf litter breakdown (Graça 2001; Hieber and Gessner 2002; Gessner et al. 2010). However, the relative importance of aquatic fungi and shredders on leaf litter breakdown in Andean streams has been poorly studied (Mathuriau and Chauvet 2002; Encalada et al. 2010), contrary to temperate (see Gessner et al. 2010 and cited literature therein), and tropical lowland streams (Boyero et al. 2015).

To understand the effects of land use on leaf litter breakdown in Andean streams, we examined how different land uses such as native forest, pasture, or urban affect in‐stream physico‐chemical variables, the composition of benthic macroinvertebrates, leaf litter‐associated macroinvertebrates and fungi, and leaf litter breakdown. We selected two catchments with a historically similar pattern of land use. In each, we set up three study sites, nowadays dominated by forest, pasture, and urban areas (a total of six sites). We predicted variations in‐stream physico‐chemical variables, such as an increase in water temperature and nutrient concentrations along the forest–pasture–urban gradient. We additionally predicted macroinvertebrate and fungal communities to have lower abundance and richness as land use changed from natural to anthropogenically disturbed conditions, and breakdown rates to differ between land uses, being fastest in impacted sites because of microbial activity stimulated by nutrient concentrations and water temperature. Finally, we assessed the in‐stream physico‐chemical variables that determine diversity of macroinvertebrates and fungi and how variation in those aquatic communities and stream physico‐chemical variables relates to breakdown rates. This information will allow us to increase the knowledge on the relationships between abiotic factors, biodiversity, and ecosystem functioning in Andean streams and provide information for the management of Andean catchments affected by land‐use change.

## Methodology

### Study sites

This study was conducted in two third‐order streams (Jipiro and El Carmen streams) of two catchments of the Zamora River basin in the southern Ecuadorian Andes between 3°56′ and 4°3′S and 79°8′ and 79°12′W. Both streams are located to the west of the city of Loja, the most populated city of southern Ecuador with around 120,000 inhabitants (Instituto Nacional de Estadísticas y Censos, 2010). We selected these streams because their catchments have a similar composition, distribution, and history of land use, as well as similar dominant soil types, namely Entisols and Inceptisols (Ochoa‐Cueva et al. 2015). Native forest, pasture, and urbanization are the main contemporary land‐cover/land‐use types (see Table S1), among these secondary native forests cover the largest area in each catchment (Iñiguez‐Armijos et al. 2014). These native forests are generally classified as montane evergreen forests (Homeier et al. 2008; Tapia‐Armijos et al. 2015). Since the 1960s, these native forests have been replaced by pasture for livestock, which is mainly concentrated in the middle and lower sections of the catchments (Torrachi et al. 2013), reaching annual deforestation rates up to 2.7% between 1989 and 2008 (Tapia‐Armijos et al. 2015). Urban development is located at the downstream part of the catchments, influencing the streams by channelization and sediment and sewage inputs.

We established three study sites characterized by different land uses (i.e., forest, pasture, and urban) at each stream (total *n *=* *6). Forest sites were located upstream, followed by pasture and then urban sites located downstream. Forest and urban sites were separated by 1.7–1.8 km with pasture sites in between. Study sites consisted of 30‐m‐long reach (riffles) with a riparian buffer of 15 m width and were located between 2050 and 2259 m a.s.l. In the forest sites, riparian vegetation presented dense canopy cover (72–78%) and were dominated by native trees of the genera *Croton*,* Hedyosmum*,* Clusia*,* Morella,* and *Juglans*. Pasture sites presented open canopy (31–34%), and the presence of trees of *Alnus acuminata* Kunth and occasionally trees of the genera *Inga* and *Eucalyptus* which were planted along the riparian margins. In the urban sites, the streams were affected by channelization on the right margin. Here, the riparian sections were mostly covered (12–12.5 m width) by impervious surface and contained a thin (2.5–3 m width) section of grassland with planted trees of *Salix* spp. and *A. acuminata* and an open canopy (24–30%) along the riparian margins. During this study (October–December 2011), the mean monthly precipitation and air temperature were 96.3 mm and 16.4°C, respectively.

### Physico‐chemical variables

On each sampling day, we measured channel width, depth, and current velocity (electromagnetic sensor FLO‐MATE 2000; Marsh‐McBirney Inc, Frederick, MD) at four to six points along four transects placed in each study site. Water temperature, specific conductance (SC), pH, and dissolved oxygen (DO) concentration were measured using a portable multisensor probe (WTW Multi 3430, Weilheim, Germany). Water samples (500 mL) were taken to analyze alkalinity, nitrate (${\text{NO}}_{3}^{-}$ ), phosphate (${\text{PO}}_{4}^{3-}$), and turbidity at the laboratory using standard methods (American Public Health Association, 1995).

### Leaf litter breakdown

We estimated leaf litter breakdown rates by applying the litter bag technique described by Bärlocher (2005). For the litter, we used alder leaves (*A. acuminata*) because this is a native species occurring frequently along the riparian zones in the studied streams. Leaves were gathered after abscission from the ground of a single location over 10 days in September 2011. In the laboratory, petioles were removed to increase homogeneity of the leaf material. Leaves were air‐dried for five days, and 4 g (SD = 0.05) of litter was enclosed within mesh bags of 16 × 17 cm.

We used two mesh sizes to evaluate leaf litter breakdown in the presence (coarse mesh bags; 10 mm mesh opening) and absence (fine mesh bags; 0.5 mm mesh opening) of macroinvertebrates. Twenty‐litter bags of each mesh size were deployed in each study site on October 2011. Litter bags of each mesh size were tied in groups of five to iron bars driven into the substrate and distributed along the study site around 10 m from one another. Four replicates of each mesh size were retrieved from each study site after 3, 14, 28, 42, and 56 days of incubation. However, in one of the urban sites (Jipiro catchment), most of the litter bags were removed by people after day 25, and thus, only one coarse and fine mesh bag were retrieved on day 28, one fine mesh bag was retrieved on day 42, and no litter bags were retrieved on day 56.

After retrieval, litter bags were placed in individual plastic bags and taken to the laboratory. Leaf material remaining was carefully rinsed with distilled water into a 500‐*μ*m mesh sieve to remove attached sediments and to recover small litter fragments, and associated macroinvertebrates in the case of coarse mesh bags. Macroinvertebrates were preserved in ethanol until being sorted and identified (see below). From each fine mesh bag, we cut two sets of eight leaf disks (20 mm diameter) for fungal determinations (see below). We did not carry out fungal determinations in coarse mesh bags because we expected same fungal activity in both mesh sizes (see Rincón and Santelloco 2009; Taylor and Chauvet 2014). We used another set of 10 leaf disks cut randomly from the leaf material to estimate the average mass of each leaf disk used for fungal determinations. Leaf material remaining and the set of 10 disks were dried at 50°C for 48 h and weighed to obtain dry mass. Dry litter was then combusted at 500°C for 4 h, and the ash was weighed. Litter ash‐free dry mass (AFDM) remaining was estimated by subtracting ash mass from dry mass (Bärlocher 2005). For fine mesh bags, the average disk AFDM was multiplied by the number of disks cut and added to the litter AFDM remaining of each bag to account for the disk mass removed for fungal determinations.

Litter AFDM remaining over time was expressed as a percentage of initial AFDM. Initial air‐dry mass was converted into initial AFDM by a conversion factor estimated from extra sets of 10 L bags of each mesh size. These extra litter bags were prepared as other samples and were exposed in the streams on day 0, recovered immediately, taken to the laboratory, and processed as described above. The conversion factor of initial air‐dry mass to initial AFDM thus included losses due to handling.

### Fungal communities

Fungal richness associated with decomposing litter was estimated from fine mesh bags by applying molecular techniques. We chose these techniques because traditional microscopy techniques can only identify fungal species sporulation, while molecular techniques can circumvent this problem (Nikolcheva et al. 2003; Nikolcheva and Bärlocher 2005). Each set of eight leaf disks was enclosed in a sterile Petri dish and frozen at −80°C until DNA extraction. DNA was isolated using an Ultraclean Soil DNA extraction kit (MoBio Laboratories, Carlsbad, CA). rDNA was amplified for the ITS region of fungi ITS1‐5.8S‐ITS2 with polymerase chain reaction (PCR) using universal primers ITS1 and ITS4 (White et al. 1990).

PCR was performed with a Phusion Master Mix (Finnzymes, Espoo, Finland) in a final volume of 20 *μ*L containing 0.5 *μ*M of forward and 0.5 *μ*M reverse primer, 1 mg mL^−1^ bovine serum albumin (BSA), 10 *μ*L of the Phusion HF master mix, and 2 *μ*L of sample DNA. All PCRs were made in a Veriti 96 Well Thermal Cycler Applied. The following program was used: (1) initial denaturation at 98°C for 30 s, (2) denaturation at 98°C for 10 s, (3) annealing at 60°C for 20 s, (4) extension at 72°C for 60 s, (5) repeat steps 2–4 for 29 cycles, (6) final extension at 72°C for 420 s, and (7) pause at 4°C until retrieved. PCR products were analyzed on a 2% agarose gel. Each PCR product was purified with the Wizard® SV Gel and PCR Clean‐Up System (Promega, Fitchburg, WI) and cloned into a Zero Blunt TOPO vector (Invitrogen, Carlsbad, CA) according to manufacturer′s protocol. Sequencing was performed in a 3500 Genetic Analyzer Applied using universal primers M13F and M13R. Nucleotide sequences were used to search for similarities within the Gen Bank and EMBL databases using BLAST algorithm (Altschul et al. 1997).

Once sequences were obtained we proceeded with species identification. To do so, an improved homology‐based analysis of the sequences was conducted (A. Sánchez‐Rodríguez, unpubl. data). Briefly, each of the obtained sequences was converted into a family of subsequences by progressively trimming 10 bp from each end until a minimum length of 200 bp was reached. All sequences in a given family (together with the original) were then used as queries in a series of BLAST searches, and the best hit was recorded. Only in those cases where the same best hit was consistently obtained for each member of a family was a species assignment made in order to reduce the number of spurious species identifications. Results were expressed as fungal richness (no. taxa sample^−1^).

The second set of eight leaf disks was used to quantify ergosterol concentration, which can be used as a surrogate of fungal biomass. Ergosterol was extracted in 10 mL KOH/methanol (8 g L^−1^) for 30 min at 80°C, then purified by the solid‐phase extraction (SPE) method and quantified with high‐pressure liquid chromatography (HPLC) by measuring absorbance at 282 nm (Gessner 2005). Also, a conversion factor of 5.5 mg ergosterol g^−1^ fungal biomass was used (Gessner and Chauvet 1993), and results were expressed as fungal biomass *μ*g g^−1^ AFDM.

### Macroinvertebrate communities

Macroinvertebrates associated with litter in coarse mesh bags were identified primarily to genus level based on Roldán Pérez (2003), but when genus identification was not possible, the family name was given. We also assigned the macroinvertebrates to functional feeding groups (FFG) according to Cummins et al. (2005 and Tomanova et al. (2005). For data analysis, results were expressed as total macroinvertebrate and shredder abundance (no. individuals sample^−1^) and richness per litter bag (no. taxa sample^−1^).

We sampled benthic invertebrate communities at each study site on day 56 to determine the community composition for each land use. Using a D‐frame dip net (0.5 mm opening mesh), we took four samples in riffles up to 0.25 m in depth by kicking the substrate for 40 s covering a sampling area of 0.30 × 1.5 m. A D‐frame dip net was used due to its suitability for sampling in a variety of stream substrates and depths (Barbour et al. 1999; Hauer and Resh 2007), contrary to the Surber sampler that is not useful for depths greater than 0.15 m. Samples were treated individually, and macroinvertebrates were preserved in ethanol and processed as described above. Results were expressed as total macroinvertebrate and shredder abundance (no. individuals sample^−1^) and richness (no. taxa sample^−1^).

### Data analysis

All statistics were performed in R environment (R Development Core Team, 2014) applying different packages mentioned below.

In order to visualize the overall differences in terms of stream characteristics between land uses, we applied a principal component analysis [PCA; “stats” package; R Development Core Team (2014)] with all stream physico‐chemical variables (Table 1). Prior to PCA, altitude, current velocity, water temperature, SC, alkalinity, ${\text{NO}}_{3}^{-}$ , and ${\text{PO}}_{4}^{3-}$ were log transformed to meet the assumptions of PCA, such as normality and to have these variables in scales comparable to that of pH and DO. In accordance with Quinn and Keough (2002), the separation among forest, pasture, and urban sites along the two most explicative axes of the PCA was assessed with an ANOVA (“stats” package). We then applied an ANOVA (“stats” package) followed by post hoc comparisons using Tukey's HSD test [“agricolae” package; de Mendiburu (2010)] on each stream physico‐chemical variable to detect individual differences between study sites. Breakdown rates for each mesh size were estimated using an exponential decay model

$$M_{t}=M_{0}e^{-kt}$$

where *M*_*t*_ is the remaining mass at time *t* (days), *M*_0_ is the initial mass, and *k* is the exponential decay coefficient or breakdown rate (Bärlocher 2005). Breakdown rates *k* were expressed per day (day^−1^), and differences between land use and mesh size were assessed by a split‐plot ANOVA, using stream as random factor, land use within stream as “between‐subject” factor, and mesh size as “within‐subject” factor. In this manner, land use (three levels) and mesh size (two levels) were treated as fixed factors. For the error terms, an appropriate random effect for each level of the split‐plot design was incorporated according to Quinn and Keough (2002). In this way, error terms were calculated by testing main factor interaction. To perform the split‐plot ANOVA, four replicates (litter bags) of *k* in each mesh size were used for each land use. We also applied a Tukey's HSD test to distinguish the effect of land use during leaf litter breakdown for both mesh sizes.

**Table 1:** Stream physico‐chemical variables (mean ± SE; *n *=* *6) in Andean stream reaches across riparian land‐use types between September and December 2011. Different superscript letters denote significant differences of means at *P *≤* *0.05 between land uses (Tukey's HSD test). For stream physico‐chemical variables, five replicates (measurements) were used for each land use. Altitude was measured once at each site

Variable	Forest	Pasture	Urban
Altitude (m a.s.l)	2,227–2,259	2,070–2,135	2,050–2,095
Width (m)	4.4 ± 0.3^a^	4.5 ± 0.2^a^	4.5 ± 0.2^a^
Depth (cm)	16.8 ± 1.2^a^	15.1 ± 1.8^a^	16.1 ± 1.7^a^
Current velocity (cm s^−1^)	54.3 ± 0.5^a^	53.9 ± 0.4^a^	58.1 ± 0.5^a^
Water temperature (°C)	13.5 ± 0.2^a^	17.0 ± 0.3^b^	17.6 ± 0.3^b^
pH	6.9 ± 0.1^a^	6.9 ± 0.1^ab^	7.1 ± 0.1^b^
SC (*μ*S cm^−1^)	26.9 ± 2.4^a^	45.3 ± 0.9^b^	59.9 ± 1.7^c^
DO (mg L^−1^)	7.9 ± 0.1^a^	7.3 ± 0.1^b^	7.2 ± 0.1^b^
Alkalinity (mg L^−1^ CaCO_3_)	16.9 ± 1.5^a^	22.2 ± 1.6^b^	26.1 ± 1.9^b^
${\text{NO}}_{3}^{-}$ (*μ*g N L^−1^)	210 ± 50^a^	1600 ± 130^b^	2500 ± 150^c^
${\text{PO}}_{4}^{3-}$ (*μ*g P L^−1^)	130 ± 10^a^	200 ± 10^b^	230 ± 20^b^
Turbidity (NTU)	0.5 ± 0.1^a^	3.7 ± 2.3^b^	14.6 ± 6.7^b^

SC, specific conductance; DO, dissolved oxygen; ${\text{NO}}_{3}^{-}$ , nitrate; ${\text{PO}}_{4}^{3-}$, phosphate.

Overall differences in benthic macroinvertebrate composition between study sites were examined by applying a permutational multivariate analysis of variance (perMANOVA; “vegan” package; Oksanen et al. 2014). Additionally, we compared total benthic macroinvertebrate and shredder abundance and richness between land use by applying an ANOVA using land use as fixed factor within stream. To perform the ANOVAs, four replicates (benthic samples) of the biological variables were used for each land use. Normal distribution of residuals was assessed by Shapiro–Wilk test, and data were log transformed as needed. Differences between study sites were also tested by post hoc comparisons using Tukey's HSD test. Then, macroinvertebrate and shredder abundance and richness and fungal richness and biomass associated with submerged leaf litter were similarly analyzed. In this case, four replicates (litter bags) of the biological variables were used for each land use.

Relationships between breakdown rates, biological (total macroinvertebrate and shredders richness and abundance and fungal richness and biomass), and stream physico‐chemical variables were examined with multiple linear regressions (MLR; “stats” package). For this analysis, macroinvertebrate variables were calculated using invertebrates from coarse mesh bags, assuming they are directly related to leaf litter breakdown. Prior to the definition of explanatory variables in MLR, PCA was used as a variable reduction technique to combine highly intercorrelated stream physical–chemical variables into independent predictors, that is, principal components. Only correlated variables were reduced into a single principal component (PC1; see Results) representing stream characteristics commonly associated to the longitudinal gradient in montane streams as mentioned in (Finn and Poff 2005). The remaining stream physical–chemical variables (nonintercorrelated) and the PC1 (hereafter longitudinal gradient) were checked for collinearity using the Pearson's correlation coefficient (“stats” package), and the nonhighly intercorrelated ones (*r *<* *60) were selected as explanatory variables (see Results). As a result of this analysis, current velocity, water temperature, pH, ${\text{PO}}_{4}^{3-}$, and the longitudinal gradient were used as predictors in the MLRs. In this manner, we retained the least statistically redundant variables and the most biologically interesting predictors considered to be important regulators of decomposer communities and organic matter breakdown in‐stream ecosystems (Mathuriau and Chauvet 2002; Rosemond et al. 2002; Dangles et al. 2004; Lujan et al. 2013).

In a first set of MLRs aimed to assess abiotic determinants of diversity of macroinvertebrates and fungi associated with leaf litter breakdown, the biological variables were treated as response variables and selected stream physical–chemical variables (see above) as explanatory ones. In a second set of MLRs aimed to determine how variation in those variables relates to leaf litter breakdown, breakdown rates *k* (day^−1^) of both mesh sizes were treated as response variables and biological and selected stream physico‐chemical variables as explanatory ones. For the MLR using breakdown rates in coarse mesh bags, we also included the abundance of the most important shredders to leaf litter breakdown in Andean streams according to Encalada et al. (2010) and to Dangles et al. (2011). Similarly as above, biological variables were checked for collinearity, nonconstant variance and log transformed as needed. To determine the best MLRs, that is, most significant model containing the predictors with the highest explanatory power, a backwards elimination technique was used to remove the least significant variables and the reduced model was re‐evaluated until it contained only significant variables. The Akaike information criterion (AIC) was used to select the best models. Then, for each of the MLRs, we applied a hierarchical partitioning analysis (“hier.part” package; Walsh and Mac Nally 2013) to examine the independent contribution (*R* ^2^) of predictors to response variables (Quinn and Keough 2002; Logan 2010). The significance of the independent contribution of predictor variables was tested by calculating Z scores at the 95% level (*Z* ≥ 1.65) after performing a randomization test (1000 times). In this manner, we estimated the relative importance (%) of each of the predictor variables in explaining fungal and macroinvertebrate diversity and breakdown rates.

## Results

### Stream characterization

Overall, stream sites differed between land use as indicated by the PCA ordination plot (Fig. S1). PCA produced two significant principle components explaining together a total of 73% of the variation in‐stream characteristics between stream sites. Forest sites were significantly separated from pasture and urban sites across PC1 axis but not across PC2 axis (ANOVA on PC1 axis: *P *<* *0.01; ANOVA on PC2 axis: *P *>* *0.06). PC1 was strongly positively associated with water temperature, SC, alkalinity, NO3¯ and turbidity, and negatively associated with altitude and DO, while PC2 was strongly positively related to current velocity and depth (Fig S1). Analysis on individual stream physico‐chemical variables supported the separation of stream sites along PC1. Channel width, depth, and current velocity did not show differences between land use (Table 1). Specific conductance, pH, and nitrate concentration were significantly higher in urban than in forest sites, with pasture sites generally presenting intermediate values (*P *<* *0.02). Turbidity, temperature, alkalinity, and phosphate concentrations were significantly higher in pasture and urban than in forest sites (*P *<* *0.01). Dissolved oxygen concentration was significantly lower in pasture and urban than in forest sites (*P *=* *0.01).

### Leaf litter breakdown

**Figure 1:**
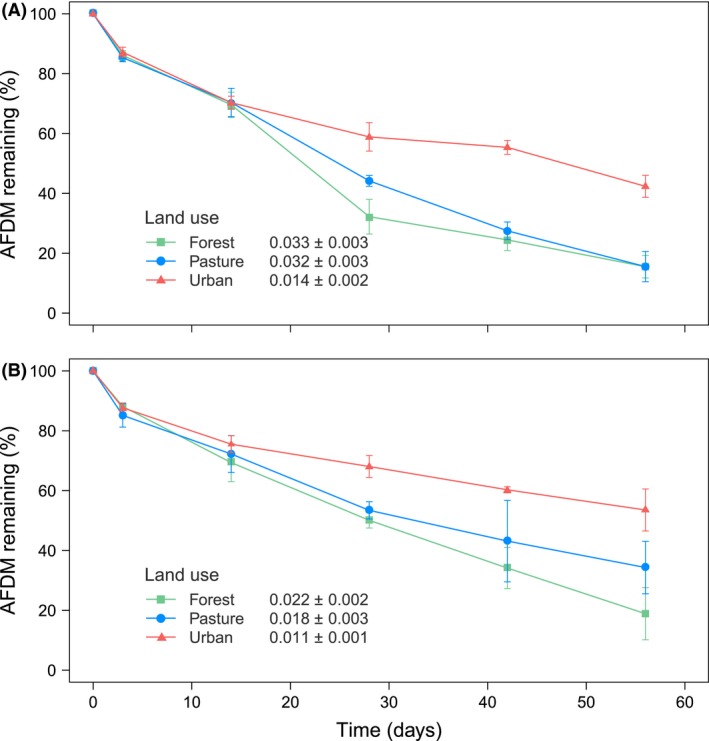
Percentage of mass remaining (mean ± SE) of alder (*Alnus acuminata*) litter incubated in coarse (A) and fine mesh bags (B) along a land‐use gradient in Andean streams over 56 days. Mean breakdown rates k (day^−1^ ± SE) for each land‐use type are shown.

During the first three days of incubation, leaching of soluble compounds caused the loss of approximately 13% of initial mass across both mesh bag types and stream sites (Fig. 1). After 56 days of incubation, coarse mesh bags lost between 58% (urban sites) and 85% (forest and pasture sites) of their initial mass, while fine mesh bags lost 46%, 66%, and 78% of their initial mass in urban, pasture, and forest sites, respectively.

**Figure 2:**
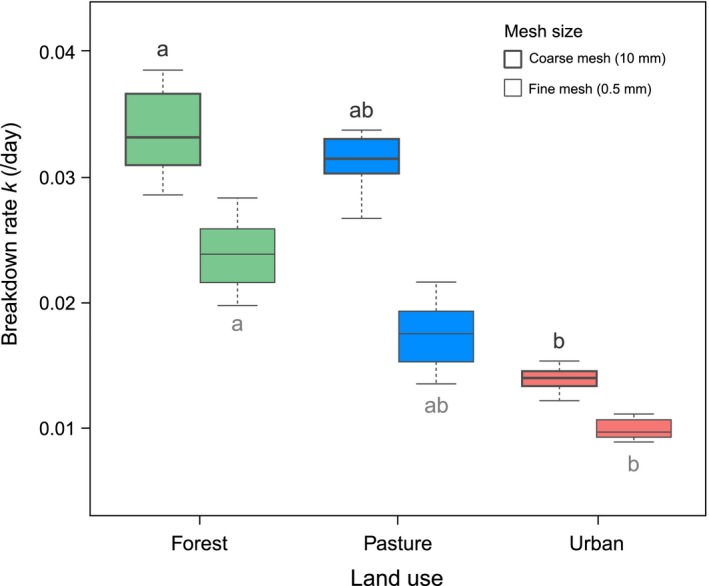
Breakdown rates of alder litter incubated in coarse and fine mesh bags along a riparian land‐use gradient in Andean streams over 56 days. Different lowercase letters denote significant differences of means at *P *≤* *0.05 between land uses (Tukey's HSD test).

Leaf litter breakdown rates did not differ significantly between streams, but rates were significantly higher in coarse mesh (average across streams and riparian land‐use type; *k *= 0.023 day^−1^) than in fine mesh bags (*k *= 0.017 day^−1^). Breakdown rates were significantly higher in forest than in urban sites, with pasture sites showing intermediate *k* values for both coarse and fine mesh bags (Fig. 2; Table 2).

**Table 2:** Summary table of the split‐plot ANOVA for breakdown rates (*k*) of alder litter in coarse and fine mesh bags (Mesh size; two levels) incubated in Andean streams along a riparian land‐use gradient (Land use; three levels). Stream was considered as random factor, land use within stream as “between‐subject” factor (fixed), and mesh size as “within‐subject” factor (fixed). Error terms were calculated by testing main factors interaction. For each mesh size, four replicates (litter bags) of *k* were used for each land use

Source of variation	df	SumSqs	MeanSqs	*F*	*P*
Block					
Residuals	1	0.00003	0.00003		
Between‐subject					
Land use	2	0.00242	0.00121	114.51	<0.01
Residuals	2	0.00002	0.00001		
Within‐subject					
Mesh size	1	0.00093	0.00093	86.36	<0.01
Land use × Mesh size	2	0.00023	0.00012	10.87	<0.01
Residuals	39	0.00041	0.00001		

### Leaf litter‐associated fungi

**Figure 3:**
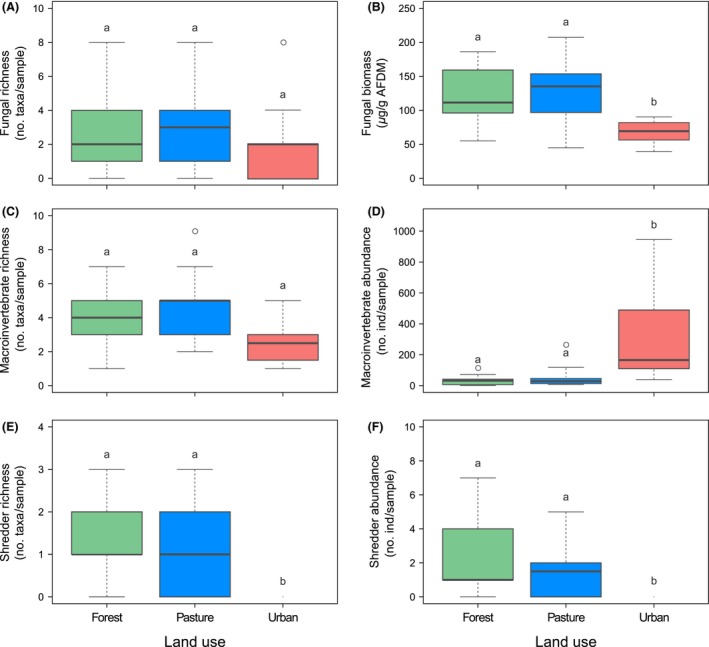
Fungal richness (A) and fungal biomass (B), total macroinvertebrate richness (C) and abundance (D), shredder species richness (E), and abundance (F) associated with alder litter incubated along a riparian land‐use gradient in Andean streams over 56 days. Different lowercase letters denote significant differences of means at *P *≤* *0.05 between land uses (Tukey's HSD test). In urban sites, shredder individuals were not present in litter bags.

Fungal species richness in fine mesh bags did not significantly differ between sites (Fig. 3A; Table 3). Ascomycota was the only fungal group found of the total species recorded. The most common fungal species found were *Articulospora tetracladia* (32%) and *Angulospora* sp. (26%). There were fungal species present in more than two land‐use types, as well as fungal species which were present in a single land‐use type (see Table S2).

**Table 3:** Summary table of the ANOVA for total macroinvertebrate richness and abundance, shredder species richness and abundance, and fungal richness and biomass associated with alder litter incubated along a riparian land‐use gradient in Andean streams over 56 days. Land use was considered as fixed factor within stream. For each biological variable, four replicates (litter bags) were used for each land use

Source of variation	Df	SumSqs	MeanSqs	*F*	*P*
Fungal richness					
Land use	2	0.606	0.303	0.321	0.73
Residuals	21	28.303	0.943		
Fungal biomass					
Land use	2	21127	10563	5.36	0.01
Residuals	21	51211	1969		
Total macroinvertebrate richness					
Land use	2	12.250	6.125	1.53	0.24
Residuals	21	84.25	4.012		
Total macroinvertebrate abundance					
Land use	2	934344	467172	3.38	0.04
Residuals	21	1623884	1381761493		
*Shredder richness*					
Land use	2	6.583	3.292	5.59	0.01
Residuals	21	12.375	0.589		
Shredder abundance					
Land use	2	3.254	1.627	5.61	0.01
Residuals	21	6.085	0.290		

Fungal biomass was significantly higher in forest and pasture sites than in urban sites (Fig. 3B, Table 3). In addition, fungal biomass increased continuously over the incubation period at forest and pasture sites, from 45 *μ*g g^−1^ AFDM on day 3–207 *μ*g g^−1^ AFDM (forest) and 171 *μ*g g^−1^ AFDM (pasture) on day 56, while it peaked on day 42 at urban sites (108 *μ*g g^−1^ AFDM) (Fig. S2).

### Leaf litter macroinvertebrates

Total macroinvertebrate richness associated with submerged litter in coarse mesh bags was significantly higher in forest and pasture sites than in urban sites, while total abundance was much lower in forest and pasture sites than in urban sites (Fig. 3C and D; Table 3). The highest abundance in forest and pasture sites occurred on day 14 with 141 and 55 individuals per sample^−1^, respectively (Fig. S3). In the case of urban sites, the highest invertebrate abundance was on day 42 with 667 individuals per sample^−1^, respectively. In general, the most abundant invertebrate families in forest sites were Simuliidae, Baetidae, Leptohyphidae, and Chironomidae, while in pasture sites the most abundant families were Chironomidae, Simuliidae, Tubificidae, Hydropsychidae, and Baetidae. In urban sites, Chironomidae represented 98% of the total abundance.

Shredder invertebrates associated with leaf litter breakdown in coarse mesh bags were only found in forest and pasture sites, and their richness and abundance were similar between these land uses (Fig. 3E and F; Table 3). The genera *Phylloicus* (54%) and *Anchytarsus* (27%) were the dominant taxa with higher abundance in forest than in pasture sites.

### Benthic macroinvertebrates

**Figure 4:**
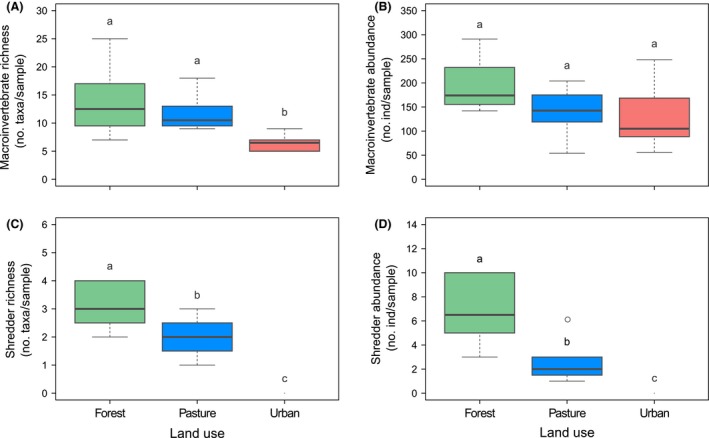
Benthic total macroinvertebrate richness (A) and abundance (B), and shredder species richness (C), and abundance (D) in Andean streams along a riparian land‐use gradient. Different lowercase letters denote significant differences of means at *P *≤* *0.05 between land uses (post hoc comparisons). In urban sites, shredder individuals were not present in D‐frame dip net samples.

Overall macroinvertebrate communities differed between sites. Macroinvertebrate assemblage in forest sites was significantly different from assemblages in pasture and urban sites, at 60% and 80%, respectively (perMANOVA; *P *<* *0.01; Table S3). No differences were found for macroinvertebrate communities between pasture and urban sites. Total macroinvertebrate richness was higher in forest and pasture sites than in urban sites, while total abundance did not significantly differ between sites (Fig 4A and B; Table 4).

**Table 4:** Summary table of the ANOVA for total benthic macroinvertebrate and shredder abundance and richness in Andean streams along a riparian land‐use gradient. Land use was considered as fixed factor within stream. For each biological variable, four replicates (benthic samples) were used for each land use

Source of variation	df	SumSqs	MeanSqs	*F*	*P*
Total macroinvertebrate richness					
Land use	2	230.58	115.292	7.27	<0.01
Residuals	21	333.25	15.869		
Total macroinvertebrate abundance					
Land use	2	19584	9792	4.11	0.03
Residuals	21	65044	3097		
Shredder richness					
Land use	2	9.108	4.554	80.15	<0.01
Residuals	21	1.1932	0.057		
Shredder abundance					
Land use	2	16.468	8.234	79.37	<0.01
Residuals	21	2.178	0.104		

Shredder richness and abundance in benthic samples were significantly different between sites (Table 4), being higher in forest than in pasture sites; no shredders were found in benthos at urban sites (Fig. 4C and B). Shredder communities were dominated by the caddisflies genera *Phylloicus* (Calamoceratidae) and *Marilia* (Odontoceridae) in forest sites (30% and 21%, respectively), while these taxa represented only 1% and 4%, respectively, in pasture sites.

### Relationships between stream physico‐chemical and biological variables

During the selection of explanatory variables for the set of MLRs assessing this relationships, PCA produced a single principal component (PC1) strongly associated with altitude, water temperature, SC, DO, alkalinity, ${\text{NO}}_{3}^{-}$ , and turbidity (Fig S1). The Pearson's correlation coefficient revealed high collinearity among these variables (*r* 0.62–0.90). With the exception of water temperature, these variables were combined into an independent predictor named longitudinal gradient which was included as a new explanatory variable. Water temperature was retained as an explanatory variable, because we were interested in assessing the effects of temperature in decomposer communities and breakdown rates. After assessing for collinearity with the remaining stream physico‐chemical variables, only water temperature showed a moderate correlation with the longitudinal gradient (*r* 0.55), and therefore, both were retained together with current velocity, pH, and ${\text{PO}}_{4}^{3-}$. Channel width and depth were omitted for further analysis because of high correlation with ${\text{PO}}_{4}^{3-}$ (*r* 0.81) and current velocity (*r* 0.68), respectively.

Associations between biological and stream physico‐chemical variables are shown in Table 5A and Table S4A. Fungal richness was significantly related to current velocity (negative relationship) and pH (negative), each explaining 35 and 40% of the variation in fungal richness, respectively. Fungal biomass was significantly associated with current velocity (negative), water temperature (negative), and phosphate concentration (negative), each contributing similarly in explaining variation in fungal biomass. Total macroinvertebrate richness was related to pH (negative) and phosphate concentration (negative), but pH explained more variation in macroinvertebrate richness than phosphate, 45 and 25%, respectively. Similarly, the total macroinvertebrate abundance was related to pH (positive), water temperature (positive), and phosphate concentration (positive). However, only the effects of pH and phosphate concentrations explained significantly 34 and 24%, respectively, of variation in macroinvertebrate abundance than water temperature (17%). Shredder richness was related to pH (negative) and phosphate concentration (negative), but only pH explained significantly the most variation (33%) in shredder richness. Shredder abundance was related to pH (negative) and water temperature (negative), but only pH explained significantly the most variation (35%) in shredder abundance.

**Table 5:** Summary of the best multiple linear regression models showing relationships between (A) biological and stream physico‐chemical variables and (B) breakdown rates *k* (day^−1^) in coarse and fine mesh bags and stream and biological variables in Andean streams. Model selection was based on Akaike information criterion (AIC). Biological variables were calculated from macroinvertebrates and fungi associated with alder litter. The symbols plus or minus in brackets indicate direction of the effect of predictor variable on response variable

Response	*R* ^2^	Total *P*	Predictor	Individual *P*
A				
Fungal richness	0.33	<0.01	Current velocity (−)	<0.01
			pH (−)	<0.01
Fungal biomass	0.50	<0.01	Current velocity (−)	0.05
			Water temperature (−)	<0.01
			${\text{PO}}_{4}^{3-}$ (−)	<0.01
Macroinvertebrate richness	0.33	<0.01	pH (−)	<0.01
			${\text{PO}}_{4}^{3-}$ (−)	0.04
Macroinvertebrate abundance	0.40	<0.01	pH (+)	<0.01
			Water temperature (+)	<0.01
			${\text{PO}}_{4}^{3-}$ (−)	<0.01
Shredder richness	0.26	<0.01	pH (−)	0.01
			${\text{PO}}_{4}^{3-}$ (−)	0.05
Shredder abundance	0.18	<0.01	pH (−)	<0.01
			Water temperature (−)	0.03
B				
Breakdown coarse	0.61	<0.01	pH (−)	<0.01
			Water temperature (−)	0.03
			${\text{PO}}_{4}^{3-}$ (−)	<0.01
			*Phylloicus* abundance (+)	0.04
Breakdown fine	0.71	<0.01	pH (−)	<0.01
			Water temperature (−)	<0.01
			Fungal biomass (+)	<0.01

### Relationships between stream physico‐chemical and biological variables and leaf litter breakdown

As it was mentioned in Methodology, we wanted to incorporate the most important shredders in the MLR for breakdown rates in coarse mesh bags, and therefore, the abundance of *Phylloicus* and *Anchytarsus* was initially considered as explanatory variables. However, the abundance of *Anchytarsus* was discarded because of its high correlation with the abundance of *Phylloicus* (*r* 0.72). Similarly, macroinvertebrate and shredder richness were discarded to be highly correlated with shredder abundance (*r* 0.63 and 0.86, respectively). As a result, both shredder and Phylloicus abundance were the only biological explanatory variables considered for breakdown rates in coarse mesh bags. In the case of breakdown rates in fine mesh bags, fungal richness and fungal biomass showed a low collinearity (*r* 0.34) and were included as predictors for these MLRs.

Relationships between leaf litter breakdown and biological and stream physico‐chemical variables were highly significant (Table 5B and Table S4B). Breakdown rates in coarse mesh bags were best predicted by pH (negative relationship), water temperature (negative), phosphate concentration (negative), and *Phylloicus* abundance (positive). However, phosphate concentration explained more variation in breakdown rates than the other significant predictors. Shredder abundance was not a significant predictor in the selected best model using AIC. Nevertheless, shredder abundance showed a similar relative contribution as *Phylloicus* abundance (≈15%) to breakdown rates in the hierarchical portioning analysis. In regard to leaf litter breakdown in fine mesh bags, a significant correlation was found with pH (negative), water temperature (negative), and fungal biomass (positive). However, pH and fungal biomass explained more variation in breakdown rates than water temperature. Fungal richness was not a significant predictor in the selected best model using AIC. Nevertheless, fungal richness showed a similar relative contribution as fungal biomass (≈26%) to breakdown rates in the hierarchical partitioning analysis.

## Discussion

In this study, we assessed the effects of the conversion of riparian native forest to pasture or urban area on Andean streams. We found that (1) land use affects stream physico‐chemical variables, for example, increasing nutrient concentration or water temperature; (2) macroinvertebrate and fungal abundance and richness are affected by current velocity, pH, water temperature, and phosphate concentration; (3) leaf litter breakdown is different between land uses, and breakdown rates became slower as riparian land‐use changes from forest to pasture to urban; and (4) leaf litter breakdown responds to pH, water temperature, phosphate concentration, fungal biomass, and the abundance of single species of leaf‐shredding invertebrates (*Phylloicus*; Trichoptera and *Anchytarsus*; Coleoptera).

Overall, we observed changes in‐stream physico‐chemical and biological variables along the riparian land‐use gradient, for example, increase in nutrient concentration or decrease in shredder abundance. But to what extent can these differences explain variations in leaf litter breakdown in Andean streams? Breakdown of alder leaf litter in both coarse and fine mesh bags responds negatively to shifts in riparian land use from natural to more disturbed, suggesting that riparian forest conversion to pasture and to urbanization reduces breakdown rates. These alterations in leaf litter breakdown along the land‐use gradient were comparable to differences observed in pH, water temperature, and phosphate concentration, and in aquatic communities between forest, pasture, and urban sites. For instance, we measured the highest pH, water temperature and phosphate concentration, the lowest shredder abundance and fungal biomass, and the lowest breakdown rates in submerged leaf litter at urban sites. These results support our hypothesis that land‐use change can modify stream physico‐chemical variables closely related with macroinvertebrate and fungal communities, in particular leaf‐shredding invertebrates and fungal activity which are responsible of leaf litter breakdown rates in Andean streams altering ecosystem functioning.

The strong effect of forest conversion on shredder invertebrates and aquatic fungi can be attributed to differences in current velocity, pH, water temperature, and phosphate concentration. Changes in these variables have been correlated with changes in decomposer communities associated with leaf litter breakdown in other temperate and tropical regions (Irons et al. 1994; Rosemond et al. 2002; Dangles and Chauvet 2003; McKie and Malmqvist 2009; McKie et al. 2009; Ferreira and Chauvet 2011; Woodward et al. 2012). In Andean streams, macroinvertebrate diversity is strongly driven by water temperature as well as by oxygen, which in turn change along the altitudinal gradient (Jacobsen et al. 1997; Jacobsen 2008; Madsen et al. 2015). Our study provides evidence that water temperature explained macroinvertebrate and shredders abundance, as well as leaf litter breakdown in coarse mesh bags. We also found that increasing water temperature showed high collinearity with decreasing dissolved oxygen and altitude, and increasing nitrate concentration along the riparian land‐use gradient, confirming that these environmental factors are usually related to the longitudinal gradient in montane streams (Finn and Poff 2005). Therefore, the changes observed in the functional assemblages of macroinvertebrate communities, particularly in leaf‐shredding invertebrates, can be also attributed to high water temperature, reduced dissolved oxygen and the presence of a more disturbed land use toward lower altitudes having additional effects on organic matter processing in Andean streams (Lujan et al. 2013).

Phosphate concentration was another significant determinant of macroinvertebrate abundance and shredder richness in our study. Both in tropical and in temperate streams, nutrient enrichment by N and P has been associated with shifts in macroinvertebrate assemblages negatively affecting shredder invertebrates and leaf litter breakdown (Rosemond et al. 2002; Woodward et al. 2012). Additionally, nutrient concentration can synergistically interact with water temperature affecting leaf litter decomposition as well (Ferreira and Chauvet 2011). We observed that shredder richness and abundance and leaf litter breakdown decreased as land‐use changes from forest to pasture to urban unlike phosphate concentration that increased along land‐use gradient. Also, breakdown rates in coarse mesh bags were explained by phosphate concentration which suggests that organic matter processing in Andean streams can be reduced by nutrient enrichment that limits to leaf‐shredding invertebrates.

With regard to fungal communities, little is known about patterns of fungal diversity and its association with abiotic factors in Andean streams. Few studies have investigated aquatic fungi related to leaf litter breakdown in these montane streams (Mathuriau and Chauvet 2002; Rincón and Santelloco 2009; Encalada et al. 2010). Those studies have shown that water temperature, current velocity, and litter quality mediate fungal activity, which in turn influence leaf litter breakdown. Here, we demonstrated that current velocity and water temperature were determinants of fungal richness and biomass, respectively, and that breakdown rates in fine mesh bags were also explained by fungal biomass and water temperature which is consistent with those findings mentioned above. We also found that fungal biomass was negatively associated to phosphate concentration, which can be explained by warmer water temperatures and increased nutrient concentration along the land‐use gradient that can reduce the availability of dissolved oxygen decreasing the activity of leaf associated fungi (Medeiros et al. 2009). For Andean streams, there is a lack of information about effects of nutrient enrichment on fungi–leaf litter breakdown relationship. Nevertheless, several studies in temperate and tropical lowland streams (Rosemond et al. 2002; Gulis and Suberkropp 2003; Woodward et al. 2012; Kominoski et al. 2014) have shown that moderate phosphorous enrichment has stimulating effects on fungal activity accelerating leaf litter breakdown in streams. In our study, the lowest fungal biomass and slowest breakdown rates were observed in urban sites unlike to phosphate concentration and water temperature that increased along the land‐use gradient showing the highest values in urban sites. These results contradict our hypothesis as we expected faster breakdown rates in impacted sites because of microbial activity stimulated by increased nutrient concentrations and water temperature. This suggests that fungal activity may be limited by high nutrient concentration inhibiting leaf litter breakdown in highly polluted Andean streams, as was found in temperate streams (Woodward et al. 2012). It is also worth noting that although fungal richness was not selected as a strong predictor of breakdown rates in fine mesh bags (Table 5B), fungal richness is likely to have contributed (Table S4B) to leaf litter breakdown along the land‐use gradient. It is particularly the case for number of fungal species and breakdown rates which were higher in forest and pasture sites than in urban sites. This finding provides indication that fungal diversity deserves further attention in future studies, because seems to be another important parameter of aquatic fungal communities in explaining microbial‐driven litter breakdown in Andean streams, contrary as mentioned in Encalada et al. (2010) who only suggest to fungal biomass as an explaining parameter.

The structure of decomposer communities can be modified by pH; for instance, low decomposer richness and slow breakdown rates (Dangles and Chauvet 2003; Dangles et al. 2004; Petrin et al. 2007) have been associated with low pH in temperate streams. These kinds of studies are limited for Andean streams. However, we have shown that fungal richness and shredder richness and abundance as well as breakdown rates both in coarse and in fine mesh bags were explained by pH, fully matching with those findings. In all study sites, the stream water was circumneutral, but pH differed slightly between land use. This suggests that small variations in pH promoted by land‐use change can synergistically interact with water temperature and phosphate concentration to reduce leaf litter breakdown in Andean streams. Therefore, more research is needed to better understand the influence of stream physico‐chemical variables in aquatic biodiversity and ecosystem functioning in these montane streams.

We believe that aquatic communities were also affected by the availability of allochthonous sources of organic matter between land uses, although we did not measure organic matter inputs and its quality. This hypothesis can be supported by the fact that the lowest fungal biomass and no shredders were observed in urban sites. Fungal activity is reduced when litter quality is low, as well as the food limitation explains the scarcity of leaf‐shredding invertebrates in tropical streams (Irons et al. 1994; Lecerf et al. 2005; Wantzen and Wagner 2006; Tomanova et al. 2007; Graça et al. 2015). Nevertheless, we have to recognize that macroinvertebrate communities were not significantly different between pasture and urban sites in the perMANOVA. Hagen et al. (2010) showed that shifts in allochthonous input to streams are dependent on land use and degradation of in‐stream conditions. In our study, we observed a decrease in riparian canopy cover from forest to urban sites and a change in tree species from native to planted species, as well as we found a decrease in fungal biomass and shredder abundance, suggesting that modifications in decomposer communities may be due to variability in organic matter input and quality. Such a change in decomposer communities has been previously observed in other Andean streams along a gradient of decomposition (Mathuriau and Chauvet 2002; Encalada et al. 2010). Our study has additionally confirmed that the negative impacts of land‐use change on stream physico‐chemical variables and decomposer communities can impair leaf litter breakdown and other functions disrupting the linkage between biodiversity and ecosystem processes of these montane streams.

### Role of shredders in leaf litter breakdown in Andean streams

Leaf litter breakdown in coarse mesh bags was significantly associated with the abundance of *Phylloicus* (Trichoptera), phosphate concentration, pH, and water temperature, while no significant associations were found with other parameters of macroinvertebrate communities. However, it is also worth noting that although shredder abundance was not selected as a strong predictor of breakdown rates (Table 5B), shredder abundance is likely to have contributed to leaf litter breakdown (Table S4B). The role of shredders in leaf litter breakdown in tropical streams is under debate (Graça et al. 2015). Several studies have reported that shredders play a minor role on leaf litter breakdown in Neotropical streams (Irons et al. 1994; Mathuriau and Chauvet 2002; Gonçalves et al. 2007), while others have found shredders to be important contributors to leaf litter breakdown (Chará et al. 2007; Encalada et al. 2010; Chará‐Serna et al. 2012). Encalada et al. (2010) showed that a single shredder species, *Phylloicus*, plays a predominant role on leaf litter breakdown in Andean streams. We also found a strong association between the abundance of *Phylloicus* and breakdown rates in this study, supporting the role of this caddisfly on the functioning of Andean streams, as well as, the importance of a keystone species on ecosystem processes (Hooper et al. 2005). In addition, we have to highlight that the abundance of *Anchytarsus* (Coleoptera) presented collinearity with the abundance of *Phylloicus*, which suggest that *Anchytarsus* can also play an important role in organic matter processing in Andean streams. Although the abundance of both genera (or total shredder abundance) was very low compared to the total macroinvertebrate biomass in coarse mesh bags, the contribution of *Phylloicus* and *Anchytarsus* on organic matter processing in these streams is essential. This supports the findings by Dangles et al. (2011), who described that breakdown rates also respond to the relative abundance of the most efficient leaf‐shredding invertebrates in Andean streams with low diversity.

### Implications for management

Our findings provide evidence that breakdown rates in Andean streams are sensitive to land‐use change as the conversion of native forests can modify stream physico‐chemical variables and aquatic communities (e.g., fungal biomass) strongly related to leaf litter breakdown. These results suggest that a proper management of catchments needs to address the effects of land‐use change on the structure and functioning of Andean streams to protect these unique ecosystems. Maintaining 70% in native vegetation in the catchment and preserving riparian corridors can protect aquatic biodiversity and ecological condition of Andean stream ecosystems (Iñiguez‐Armijos et al. 2014) and will help maintain the ecosystem processes such as leaf litter breakdown. However, many Andean countries are affected by a high forest conversion rate (Armenteras et al. 2003; Tapia‐Armijos et al. 2015), making it difficult to promote whole‐catchment management or restoration in large regions. Riparian management as a kind of buffer control can be a means to mitigate the negative effects that these human activities (e.g., nutrient enrichment) have on aquatic communities and ecosystem processes in tropical streams (Lorion and Kennedy 2009). Nevertheless, the function of riparian buffers is subject to spatial scale patterns, land‐use intensification (Allan 2004), and terrestrial/aquatic linkages (Hladyz et al. 2011). Thus, further research will be required to determine suitable riparian buffers to promote ecological integrity and functioning of Andean streams with intensive land use.

## Conflict of Interest

None declared.

## Supporting information


**Figure S1.** Principal Component Analysis (PCA) of stream physico‐chemical variables measured along a riparian land‐use gradient in Andean streams.

**Figure S2.** Ergosterol concentration associated with alder litter in fine mesh bags incubated along a land use gradient in Andean streams over 56 days.

**Figure S3.** Macroinvertebrate abundance associated with alder litter in coarse mesh bags incubated along a land use gradient in Andean streams over 56 days.

**Table S1.** Total area and percentage of each land‐use type of the Jipiro and El Carmen catchment.

**Table S2.** List of fungal species associated with alder litter incubated along a riparian land‐use gradient in Andean streams over 56 days.

**Table S3.** Results of permutational multivariate analysis of variance (perMANOVA) of benthic macroinvertebrate communities along a riparian land‐use gradient in Andean streams.

**Table S4.** Summary of the multiple linear regression models and hierarchical partitioning showing relationships between (A) biological and stream physic‐chemical variables and (B) breakdown rates *k* (d^−1^) in coarse and fine mesh bags and stream and biological variables in Andean streams.
