# Supplementary figures and images for: Shifts in leaf litter breakdown along a forest–pasture–urban gradient in Andean streams
Source: Ecol Evol. 2016 Jun 17;6(14):4849–65. doi: 10.1002/ece3.2257 (PMC4979712; doi:10.1002/ece3.2257)

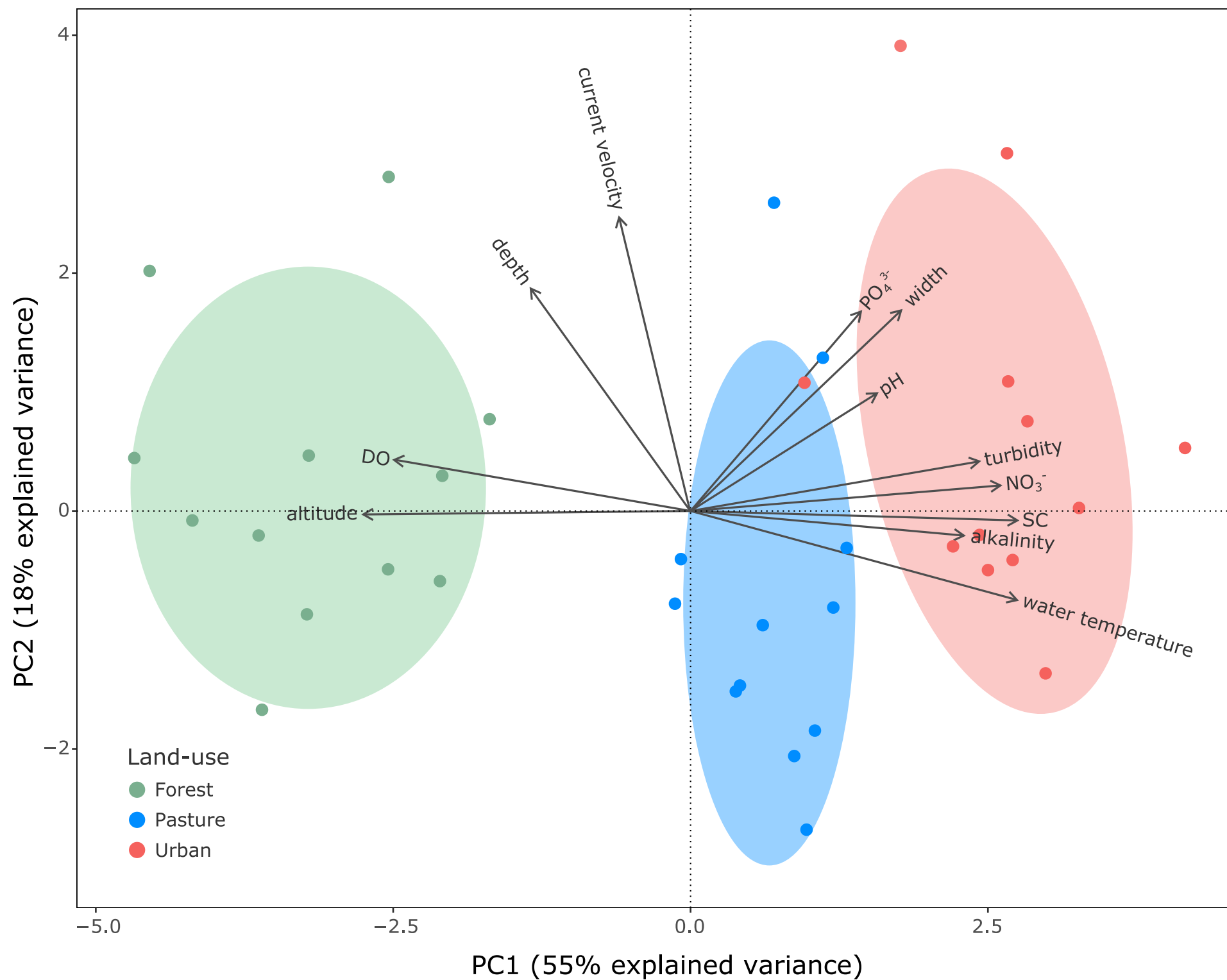

Supplement: Supplementary file 1 — Figure S1. Principal Component Analysis (PCA) of stream physico‐chemical variables measured along a riparian land‐use gradient in Andean streams. [file ECE3-6-4849-s001.pdf]

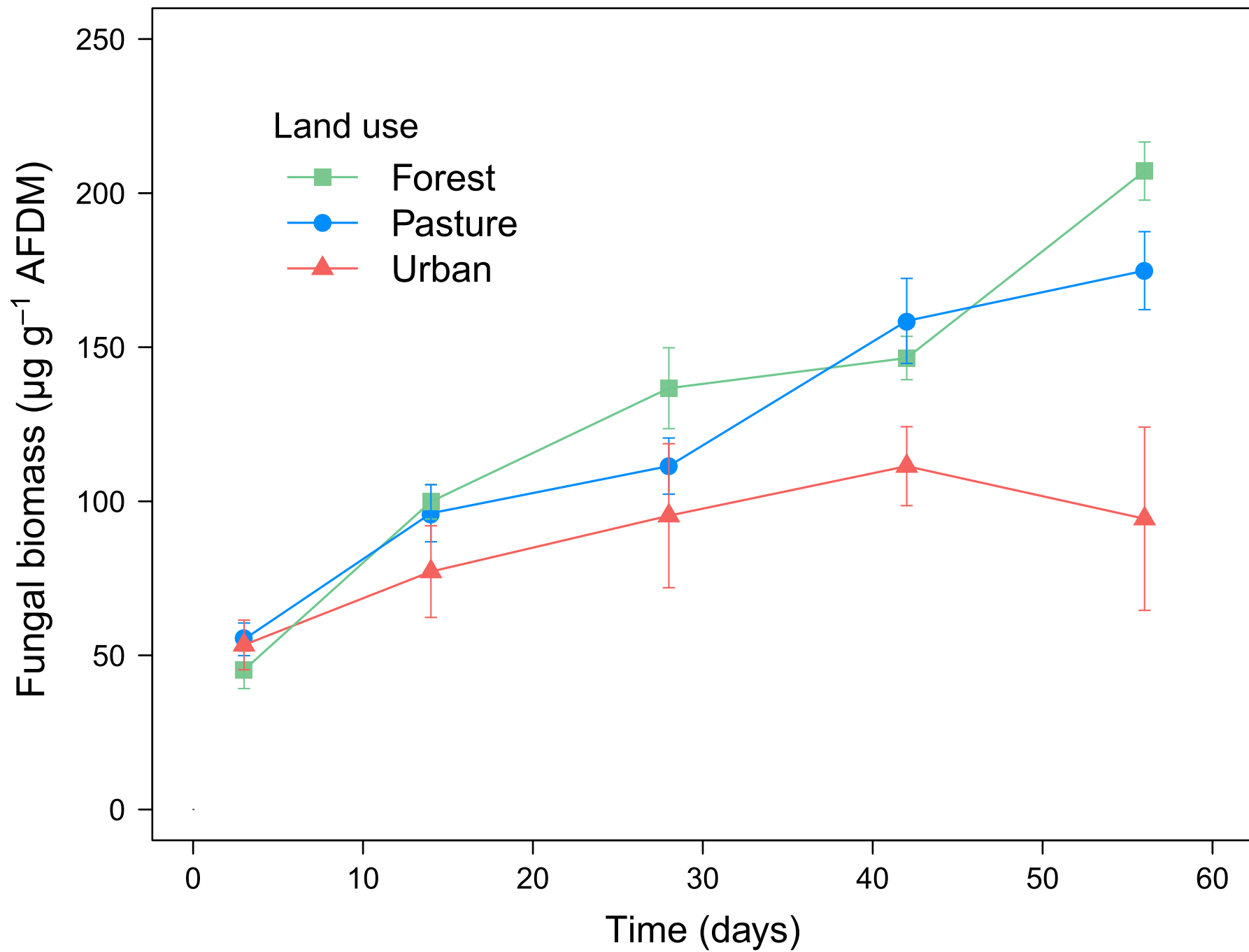

Supplement: Supplementary file 2 — Figure S2. Ergosterol concentration associated with alder litter in fine mesh bags incubated along a land use gradient in Andean streams over 56 days. [file ECE3-6-4849-s002.pdf]

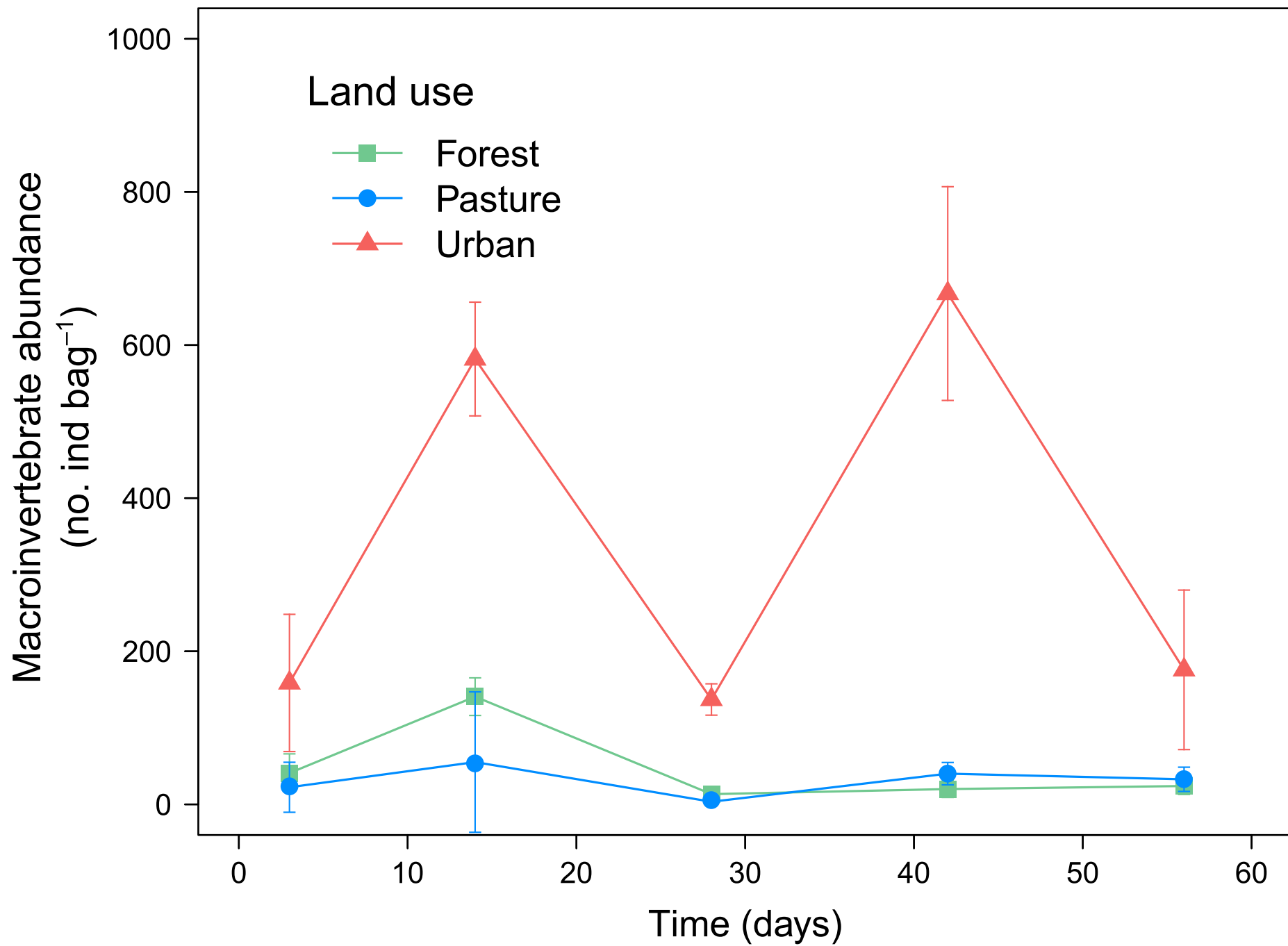

Supplement: Supplementary file 3 — Figure S3. Macroinvertebrate abundance associated with alder litter in coarse mesh bags incubated along a land use gradient in Andean streams over 56 days. [file ECE3-6-4849-s003.pdf]
