# Supplementary material for: Shifts in leaf litter breakdown along a forest–pasture–urban gradient in Andean streams
Source: Ecol Evol. 2016 Jun 17;6(14):4849–65. doi: 10.1002/ece3.2257 (PMC4979712; doi:10.1002/ece3.2257)
Supplement: Supplementary file 4 — Table S1. Total area and percentage of each land‐use type of the Jipiro and El Carmen catchment. [file ECE3-6-4849-s004.docx]

| **Table S1** Total area and percentage of each land-use type of the Jipiro and El Carmen catchment. | | | |
| --- | --- | --- | --- |
|  | Jipiro | El Carmen |  |
| Catchment size (km2) | 33.28 | 38.51 |  |
| Forest (%) | 70.98  ± 0.3 **^a^** | 71.63  ± 0.2 **^a^** |  |
| Páramo (%) | 0.94  ± 1.2 **^a^** | 3.40  ± 1.8 **^a^** |  |
| Crops (%) | 0.06  ± 0.5 **^a^** | 0.08  ± 0.4 **^a^** |  |
| Pasture (%) | 24.33  ± 0.2**^a^** | 21.66  ± 0.3**^b^** |  |
| Urban (%) | 3.69  ± 0.1**^a^** | 3.23  ± 2.3**^b^** |  |
