# Supplementary material for: Shifts in leaf litter breakdown along a forest–pasture–urban gradient in Andean streams
Source: Ecol Evol. 2016 Jun 17;6(14):4849–65. doi: 10.1002/ece3.2257 (PMC4979712; doi:10.1002/ece3.2257)
Supplement: Supplementary file 5 — Table S2. List of fungal species associated with alder litter incubated along a riparian land‐use gradient in Andean streams over 56 days. [file ECE3-6-4849-s005.docx]

| **Table S2** List of fungal species associated with alder litter incubated along a riparian land-use gradient in Andean streams over 56 days | | | | |
| --- | --- | --- | --- | --- |
| Phylum | Species | Forest | Pasture | Urban |
| Ascomycota | *Anguillospora filiformis* |  | X | X |
|  | *Angulospora* spp. | X | X | X |
|  | *Articulospora* spp. |  | X |  |
|  | *Articulospora tetracladia* | X | X | X |
|  | *Cladosporium* spp. |  | X |  |
|  | *Clavariopsis aquatica* |  |  | X |
|  | *Hydrocina chaetocladia* | X | X |  |
|  | *Hymenoscyphus* spp. | X |  | X |
|  | *Monographella lycopodina* |  | X |  |
|  | *Monographella nivalis* | X |  |  |
|  | *Tetrachaetum elegans* | X | X | X |
|  | *Tricladium biappendiculatum* | X |  |  |
|  | *Varicosporium elodeae* | X |  |  |
|  | No. of taxa | 8 | 8 | 6 |
