# Supplementary material for: Shifts in leaf litter breakdown along a forest–pasture–urban gradient in Andean streams
Source: Ecol Evol. 2016 Jun 17;6(14):4849–65. doi: 10.1002/ece3.2257 (PMC4979712; doi:10.1002/ece3.2257)
Supplement: Supplementary file 6 — Table S3. Results of permutational multivariate analysis of variance (perMANOVA) of benthic macroinvertebrate communities along a riparian land‐use gradient in Andean streams. [file ECE3-6-4849-s006.docx]

| **Table S3** Results of permutational multivariate analysis of variance (perMANOVA) of benthic macroinvertebrate communities along a riparian land-use gradient in Andean streams. Dissimilarity percentage according to Bray-Curtis distance | | |
| --- | --- | --- |
| Land use | % Dissimilarity | *p* |
| Forest *≠* Pasture | 60.0 | <0.01 |
| Forest *≠* Urban | 80.5 | <0.01 |
| Pasture *≠* Urban | 45.3 | 0.32 |
