# Supplementary material for: Shifts in leaf litter breakdown along a forest–pasture–urban gradient in Andean streams
Source: Ecol Evol. 2016 Jun 17;6(14):4849–65. doi: 10.1002/ece3.2257 (PMC4979712; doi:10.1002/ece3.2257)
Supplement: Supplementary file 7 — Table S4. Summary of the multiple linear regression models and hierarchical partitioning showing relationships between (A) biological and stream physic‐chemical variables and (B) breakdown rates k (d−1) in coarse and fine mesh bags and stream and biological variables in Andean streams. [file ECE3-6-4849-s007.docx]

| **Table S4** Summary of the multiple linear regression models and hierarchical partitioning showing relationships between (A) biological and stream physic-chemical variables and (B) breakdown rates *k* (d^–1^) in coarse and fine mesh bags and stream and biological variables in Andean streams. Biological variables were calculated from macroinvertebrates and fungi associated with alder litter. Longitudinal gradient is a principal component (PC1) associated with altitude, dissolved oxygen, specific conductance, nitrate concentration, alkalinity and turbidity. Variables in bold were selected in best model based on Akaike Information Criterion (AIC). Z scores in bold indicate significance of predictors after performing a hierarchical partitioning analysis to assess the relative importance (Contribution %) of each of the predictor variables in explaining the response variable. | | | | |  |
| --- | --- | --- | --- | --- | --- |
| *Response variable*  Predictor variables | Independent *R*^2^ | Total *R*^2^ | Contribution % | Z score |  |
| **A** |  |  |  |  |  |
| *Fungal richness* |  |  |  |  |  |
| **Current velocity** | 0.14 | 0.21 | 34.47 | **2.70** |  |
| Water temperature | 0.04 | 0.04 | 9.39 | 0.24 |  |
| **pH** | 0.16 | 0.16 | 39.88 | **3.22** |  |
| PO_4_^3¯^ | 0.04 | 0.04 | 8.70 | 0.13 |  |
| Longitudinal gradient | 0.03 | 0.00 | 7.56 | –0.03 |  |
| *Fungal biomass* |  |  |  |  |  |
| **Current velocity** | 0.13 | 0.18 | 23.07 | **2.39** |  |
| **Water temperature** | 0.13 | 0.02 | 24.20 | **2.57** |  |
| pH | 0.08 | 0.13 | 15.25 | 1.44 |  |
| **PO_4_^3¯^** | 0.12 | 0.02 | 22.28 | **2.48** |  |
| Longitudinal gradient | 0.08 | 0.14 | 15.20 | 1.40 |  |
| *Total macroinvertebrate richness* |  |  |  |  |  |
| Current velocity | 0.06 | 0.09 | 13.72 | 0.57 |  |
| Water temperature | 0.02 | 0.00 | 5.67 | –0.18 |  |
| **pH** | 0.20 | 0.27 | 45.17 | **3.86** |  |
| **PO_4_^3¯^** | 0.11 | 0.17 | 25.23 | **2.10** |  |
| Longitudinal gradient | 0.04 | 0.05 | 10.21 | 0.32 |  |
| *Total macroinvertebrate abundance* |  |  |  |  |  |
| Current velocity | 0.06 | 0.14 | 12.03 | 0.79 |  |
| **Water temperature** | 0.09 | 0.03 | 16.87 | 1.20 |  |
| **pH** | 0.17 | 0.11 | 33.45 | **2.99** |  |
| **PO_4_^3¯^** | 0.12 | 0.06 | 24.24 | **2.15** |  |
| Longitudinal gradient | 0.07 | 0.08 | 13.41 | 0.97 |  |
| *Shredder richness* |  |  |  |  |  |
| Current velocity | 0.05 | 0.03 | 14.31 | 0.44 |  |
| Water temperature | 0.03 | 0.02 | 9.61 | 0.09 |  |
| **pH** | 0.11 | 0.19 | 33.27 | **2.07** |  |
| **PO_4_^3¯^** | 0.06 | 0.14 | 18.92 | 0.82 |  |
| Longitudinal gradient | 0.08 | 0.11 | 23.89 | 1.28 |  |
| *Shredder abundance* |  |  |  |  |  |
| Current velocity | 0.06 | 0.04 | 16.89 | 0.85 |  |
| **Water temperature** | 0.08 | 0.11 | 22.78 | 1.31 |  |
| **pH** | 0.12 | 0.21 | 35.19 | **2.25** |  |
| PO_4_^3¯^ | 0.05 | 0.12 | 15.92 | 0.67 |  |
| Longitudinal gradient | 0.03 | 0.02 | 9.22 | 0.04 |  |
| **B** |  |  |  |  | |
| *Breakdown coarse* |  |  |  |  | |
| Current velocity | 0.04 | 0.13 | 5.49 | 0.22 | |
| **Water temperature** | 0.11 | 0.03 | 15.91 | **2.20** | |
| **pH** | 0.09 | 0.02 | 13.48 | **1.66** | |
| **PO_4_^3¯^** | 0.21 | 0.37 | 30.62 | **4.27** | |
| Longitudinal gradient | 0.03 | 0.08 | 0.08 | 0.02 | |
| Shredder abundance | 0.10 | 0.27 | 14.63 | **1.79** | |
| ***Phylloicus* abundance** | 0.10 | 0.18 | 15.16 | **1.93** | |
| *Breakdown fine* | 0.15 | 0.30 | 20.02 | **2.90** | |
| Current velocity | 0.06 | 0.17 | 7.65 | 0.69 | |
| **Water temperature** | 0.07 | 0.04 | 9.71 | 1.15 | |
| **pH** | 0.15 | 0.30 | 20.02 | **2.90** | |
| PO_4_^3¯^ | 0.03 | 0.08 | 3.99 | 0.03 | |
| Longitudinal gradient | 0.05 | 0.01 | 7.00 | 0.47 | |
| Fungal richness | 0.19 | 0.44 | 25.20 | **3.04** | |
| **Fungal biomass** | 0.20 | 0.48 | 26.43 | **3.82** | |
