# Supplementary material for: Shifts in leaf litter breakdown along a forest–pasture–urban gradient in Andean streams
Source: Ecol Evol. 2016 Jun 17;6(14):4849–65. doi: 10.1002/ece3.2257 (PMC4979712; doi:10.1002/ece3.2257)
Supplement: Supplementary file 8 [file ECE3-6-4849-s008.docx]

**Supporting Information Legends**

**Fig. S1** Principal Component Analysis (PCA) of stream physico-chemical variables measured along a riparian land-use gradient in Andean streams. The ordination plot indicate a separation of stream characteristics between land-use along axis 1, but not along axis 2. Forest, pasture and urban sites were significantly different along axis 1(ANOVA on PCA axis 1; p<0.01). Arrows indicate vector fitting of stream physico-chemical variables with axes. SC= specific conductance, DO= dissolved oxygen, NO_3_^¯^= nitrate, PO_4_^3¯^= phosphate

**Fig. S2** Ergosterol concentration associated with alder litter in fine mesh bags incubated along a land use gradient in Andean streams over 56 days. Mean ergosterol concentrations (µg g^–1^ AFDM ± SE) for each land use type are shown

**Fig. S3** Macroinvertebrate abundance associated with alder litter in coarse mesh bags incubated along a land use gradient in Andean streams over 56 days. Mean number of individuals (no. ind bag^–1^ ± SE) for each land use type are shown
